# Association of indications for mandibular third molar coronectomy and the Pell and Gregory and the Winter classification systems

**DOI:** 10.1007/s10006-024-01222-5

**Published:** 2024-02-09

**Authors:** Rashida N. Simons, Mitchell S. Gonesh, Jacco G. Tuk, Jan de Lange, Jerome A. Lindeboom

**Affiliations:** 1 Department of Oral and Maxillofacial Surgery, Amsterdam University Medical Center, University of Amsterdam, Meibergdreef 9, 1105 AZ Amsterdam, the Netherlands; 2 Department of Oral and Maxillofacial Surgery, Amstelland Hospital, Amstelveen, The Netherlands

**Keywords:** Coronectomy, Surgical removal, Mandibular third molar

## Abstract

**Purpose:**

The purpose of this study was to determine how the surgeon’s decision to perform a mandibular third molar coronectomy or surgical removal is associated with the impaction pattern as classified using Pell and Gregory or Winter’s system.

**Methods:**

This observational, cross-sectional study was conducted on 813 mandibular third molars belonging to 565 patients. All patients were referred for removal of the mandibular third molar and had radiographic signs indicating a close relationship with the inferior alveolar nerve. Panoramic images were classified according to the impaction pattern.

**Results:**

A coronectomy was performed on 492 (60.5%) mandibular third molars.

Most impacted mandibular third molars were class IIB with a mesioangular inclination. A significant association was found between the Pell and Gregory classification and the surgeon’s choice (*p* = 0.002). Winter’s classification was not significantly associated with surgeon choice (*p* = 0.425).

**Conclusion:**

Mandibular third molar coronectomy is chosen more frequently than surgical removal if molars are class III and position B.

**Trial registration number:**

Not applicable.

## Introduction

Damage to the inferior alveolar nerve (IAN) is one of the most problematic complications of mandibular third molar surgical removals [1]. Injury to the IAN generally causes paresthesia or anesthesia of the lower lip, buccal gingiva, or chin area [2]. The reported incidence of temporary postoperative IAN dysfunction after removal of an impacted or erupted mandibular third molar ranges from 0.35% to 8.4% [3]. The incidence of persistent IAN involvement, defined as IAN disturbance still present at 6 months after surgery, varies from 0 to 2% [3–7]. Permanent IAN injury has been linked to a lower quality of life and multiple symptoms associated with depression [8].

Coronectomy is an alternative to complete surgical removal of an impacted mandibular third molar and can offer a way to reduce risk of IAN damage when the molar roots lie close to the IAN [9]. A coronectomy involves removal only of the crown of the mandibular third molar, with the root complex left behind in the alveolar bone, avoiding iatrogenic injury to the IAN.

Assessment of mandibular third molar position is routinely performed with panoramic radiographs. On these images, radiographic signs that are associated with a high risk of IAN exposure and injury during mandibular third molar surgery include diversion or narrowing of the mandibular canal, deflection or narrowing of the roots, loss or interruption of the white line, and a periapical radiolucent area [10, 11]. Preoperative panoramic images also can be used to determine the relative difficulty that can be expected during surgical removal of impacted third molars. The Pell and Gregory and the Winter classifications are frequently used to classify mandibular third molars by impaction pattern [12]. The Pell and Gregory classification assesses the pattern of lower third molars based on their position in the alveolar bone, the available space between the second molar and the ascending ramus of the mandible (class I/II/III), and the impaction depth of the third molar (position A/B/C) [13]. Winter’s classification relies on the inclination of the third molar relative to the long axis of the adjacent second molar [14].

Few studies have investigated the position of mandibular third molars relative to the IAN in the different categories of the Pell and Gregory system. The apex of horizontally and distoangular impacted lower third molars is more likely to be near the IAN [15, 16]. Moreover, third molars mostly embedded in the mandible ramus or impacted below the cervical line of the second molar are associated with a higher risk of IAN damage during surgical removal [16, 17]. Information remains limited regarding the Pell and Gregory and the Winter classifications and the indication for coronectomy. A better understanding of these associations could aid in selecting an appropriate surgical procedure and potentially decrease risk of IAN injury.

In the present study, the aim was to assess the relationship between the decision to perform a mandibular third molar coronectomy or surgical removal and the impaction pattern classified using either the Pell and Gregory or Winter classification system.

## Materials and methods

### Ethical considerations

This observational cross-sectional study was reviewed and approved by the Ethics Committee of the Academic Center for Dentistry Amsterdam and was documented under the registration number 2022–40429. The study was conducted in accordance with Good Clinical Practices and the Declaration of Helsinki, as amended in Somerset West, Republic of South Africa, in 1996.

### Eligible patients

Included patients were referred to the Oral and Maxillofacial Surgery Department of Amstelland Hospital, in Amstelveen, The Netherlands, for surgical removal of a mandibular third molar from January 2019 to January 2021. Digital panoramic radiographs were made with an Orthopantomograph® OP100 D (GE Healthcare, Dental, Tuusula, Finland) at a clinically standard resolution. During the scanning procedure, the Frankfort horizontal plane of the patient was set parallel to the floor, and the midsagittal plane was aligned with the long axis of the Orthopantomograph®.

Patients were eligible for this study if their images had at least one of the five radiographic signs showing close proximity of the third molar root to the IAN on preoperative panoramic images [18]: diversion of the canal (when the mandibular canal crosses the third molar and shifts in direction); narrowing of the canal, i.e., one or both of the radio-opaque lines representing the mandibular canal narrowed abruptly around the third molar; narrowing of the root, i.e., when crossing the mandibular canal, the root narrowed abruptly; interruption of the white line, i.e., when the mandibular canal was projected as two radio-opaque lines and one or both lines suddenly disappeared around the third molar; and darkening of the root because of a decrease in root density resulting from an impingement between the mandibular canal and the root of the third molar.

All eligible patients underwent additional computed tomography (CT) imaging (Philips Ingenuity 128 CT Scanner, Integrity Medical, Fort Myers, FL, USA). An experienced oral and maxillofacial surgeon and an independent radiologist evaluated the images for contact between the impacted mandibular third molar and the IAN canal. When the CT scan confirmed contact, coronectomy was performed. If the roots of the mandibular third molar did not lie near the IAN canal, surgical removal was performed.

Study exclusion criteria were no signs of proximity to the IAN on preoperative panoramic radiographs, age < 18 years, and no treatment administered after CT scan review (i.e., “watchful monitoring”).

### Study variables

The independent variable was the impaction pattern of the lower third molar. This pattern was assessed on preoperative orthopantomogram images using the Pell and Gregory and the Winter classifications (Table 1). The Pell and Gregory classification combines two categories, and we investigated the combination and each category separately. The dependent variable was the surgeon’s decision to perform mandibular third molar coronectomy or surgical removal. This variable was dichotomous and scored as surgical removal (S) or coronectomy (C).

**Table 1:** Pell and Gregory and Winter classification systems

**Pell and Gregory classification**				
• Assessment of the available space between the distal part of the second molar and the anterior border of the ascending ramus of the mandible				
Class I	Sufficient space to accommodate the mesio-distal diameter of the third molar			
Class II	Insufficient space to accommodate the mesio-distal diameter of the third molar			
Class III	Nearly all the third molar is in the mandible			
• Impaction depth				
Position A	The highest portion of the third molar lies above the occlusal plane of the second molar			
Position B	The highest portion of the third molar lies between the occlusal plane and cement–enamel junction of the second molar			
Position C	The highest portion of the third molar lies below the cement–enamel junction of the second molar			
**Winter classification**				
Inclination				
Vertical	Horizontal	Mesio-angular	Disto-angular	Other

### Evaluation of the panoramic images

A single reviewer assessed the panoramic images. To standardize viewing conditions, images were evaluated in a darkened room on a single computer monitor, without adjustment of contrast and brightness. The classification order was random, and each mandibular third molar was independently classified by the Pell and Gregory classification system and the Winter classification system. If needed, magnification and an angle measurement tool were used. To prevent visual fatigue and eye strain, a maximum of 100 images per day were evaluated.

At 2 months after the first assessment, 20% of the panoramic radiographs were randomly reassessed to measure intra-examiner reliability, determined using Cohen’s kappa. An experienced oral and maxillofacial surgeon and one independent radiologist assessed the choice of complete surgical removal versus coronectomy of the lower third molar. The surgeon made the final decision and performed the selected surgical procedure.

### Statistical analysis

A Chi-square test was used to determine if there was an overall association between the Pell and Gregory and the Winter classification systems of the mandibular third molar and the surgeon’s choice of mandibular third molar coronectomy or surgical removal. A post hoc analysis was used for statistically significant results to determine the origin of the differences. Intra-rater reliability kappa (*k*) values were calculated for each classification section. A *k* value < 0.20 was considered poor agreement, 0.21–0.40 fair agreement, 0.61–0.80 good agreement, and 0.81–1.00 very good agreement. All statistical analyses were performed using the Statistical Package for the Social Sciences, (SPSS Inc. version 26.0 IBM Inc., Armonk, NY, USA). Probability values < 0.05 were considered statistically significant. The significance level of the post hoc analysis was adjusted using Bonferroni correction.

## Results

### Intra-rater reliability

The intra-rater reliability after the 2-month interval was considered good to very good. Kappa values were calculated for each classification system, and each category of the Pell and Gregory classification was calculated independently. The kappa value for space between the second molar and ramus was 0.789, and the value for assessment of impaction depth was 0.765. The highest value of agreement, 0.861, was found for the inclination assessments.

### Summary statistics

The study sample consisted of 813 mandibular third molars in 565 patients (368 females and 197 males; mean age 27.78 ± 10.4 years, range 18–92 years; Table 2). A coronectomy was indicated in 492 (60.5%) of the 813 molars and surgical removal in 321 (39.5%).

**Table 2:** Summary of descriptive statistics

Variables	N (%)*
Number of patients	565
Number of mandibular third molars	813
Mean age (± standard deviation)	27.87 ± 10.4
Sex	
Male	280 (34.4)
Female	533 (65.6)
Surgeon’s assessment	
Surgical removal	321 (39.5)
Coronectomy	492 (60.5)
Pell and Gregory classification	
1A	23 (2.8)
1B	9 (1.1)
1C	3 (0.0)
2A	362 (44.5)
2B	320 (39.4)
2C	26 (3.2)
3A	24 (3.0)
3B	39 (4.8)
3C	7 (1.0)
Space between the second molar and the ramus	
I	35 (4.3)
II	708 (87.1)
III	70 (8.6)
Impaction depth	
A	409 (50.3)
B	368 (45.3)
C	36 (4.4)
Inclination (Winter classification)	
Horizontal	135 (16.6)
Mesioangular	370 (45.5)
Vertical	221 (27.2)
Distoangular	82 (10.1)
Other	5 (0.6)

^*^Unless otherwise indicated.

According to the Pell and Gregory system, 23 (2.8%) molars were classified as 1A, 9 (1.1%) as 1B, 3 (0.03%) as 1C, 362 (44.5%) as 2A, 320 (39.4%) as 2B, 26 (3.2%) as 2C, 24 (3.0%) as 3A, 39 (4.8%) as 3B, and 7 (1.0%) as 3C. Of the 813 mandibular third molars, 35 (4.3%) were classified as class I, 708 (87.1%) as class II, and 70 (8.6%) as class III. A, B, or C reflects an increasing degree of impaction depth. In this study sample, 409 (50.3%) were in position A, 368 (45.3%) were in position B, and 36 (4.4.%) were in position C. Based on the Winter classification, 135 (16.6%) third molar impactions were horizontal, 370 (45.5%) were mesioangular, 221 (27.2%) were vertical, 82 (10.1%) were distoangular, and 5 (0.6%) were classified as other, i.e., buccolingual or inverted (Table 2).

The Chi-square test revealed a significant association between the surgeon’s decision and the Pell and Gregory classification system (X^2^ (8) = 23.977, *p* = 0.002; Table 3). The two most common coronectomy indications were impaction patterns 3B (82.1%) and 3C (85.7%). The surgeon’s choice and the space between the second molar and the ramus also were significantly associated (X^2^ (2) = 10.471, *p* = 0.005; Table 4). Indications for a coronectomy were most frequent among molars in class III (78.6%). The association between the surgeon’s decision and the impaction depth also was significant (X^2^ (2) = 14.463, *p* = 0.001; Table 5). The indication for coronectomy was most frequent for molars in position B (67.4%), where the molar lies below the occlusal plane but above the cement–enamel junction of the adjacent second molar. The mesioangular (63.5%) inclination was the most common in the coronectomy group, but without a significant association between the surgeon’s choice and the Winter classification system (X^2^ (4) = 3.862, *p* = 0.425; Table 6).

**Table 3:** Frequency of the surgeon’s choice by different classifications of Pell and Gregory

	1A	1B	1C	2A	2B	2C	3A	3B	3C	Chi-square (df)	*P* value
Surgeon’s choice										23.977 (8)	0.002*
Surgical removal	11 (47.8)	2 (22.2)	1 (33.3)	170 (47.0)	111 (34.7)	11 (42.3)	7 (29.1)	7 (17.9)	1 (14.3)		
Coronectomy	12 (52.1)	7 (77.8)	2 (66.7)	192 (53.0)	209 (65.3)	15 (57.7)	17 (70.8)	32 (82.1)	6 (85.7)		
Total	23 (100)	9 (100)	3 (100)	362 (100)	320 (100)	26 (100)	24 (100)	39 (100)	7 (100)		

^*^Statistically significant difference at *p* < 0.05.

**Table 4:** Frequency of the surgeon’s choice by different spacing between the second molar and the ramus

	Class I	Class II	Class III	Chi-square (df)	*P* value
Surgeon’s choice				10.471 (2)	0.005*
Surgical removal	14 (40.0)	292 (41.2)	15 (21.4)		
Coronectomy	21 (60.0)	416 (58.8)	55 (78.6)		
Total	35 (100)	708 (100)	70 (100)		

^*^Statistically significant difference at *p* < 0.05.

**Table 5:** Frequency of the surgeon’s choice by different impaction depths

	Position A	Position B	Position C	Chi-square (df)	*P* value
Surgeon’s choice				14.463 (2)	0.001*
Surgical removal	188 (46.0)	120 (32.6)	13 (36.1)		
Coronectomy	221 (54.0)	248 (67.4)	23 (63.9)		
Total	409 (100)	368 (100)	36 (100)		

^*^Statistically significant difference at *p* < 0.05.

**Table 6:** Frequency of the surgeon’s choice at different inclinations

	Horizontal	Mesioangular	Vertical	Distoangular	Other	Chi-square (df)	*P* value
Surgeon’s choice						3.862 (4)	0.425*
Surgical removal	55 (40.7)	135 (36.5)	90 (40.7)	39 (47.6)	2 (40.0)		
Coronectomy	80 (59.3)	235 (63.5)	131 (59.2)	43 (52.4)	3 (60.0)		
Total	135 (100)	370 (100)	221 (100)	82 (100)	5 (100.0)		

^*^Statistically significant difference at *p* < 0.05.

Post-hoc analyses revealed that a coronectomy was more frequently indicated for molars in class III (78.6%) compared to molars in class II (58.9%; *p* = 0.001; Table 7). Thus, for mandibular third molars classified as class III, the surgeon tended to conduct coronectomy, and for those classified as class II, surgical removal was chosen. The frequency of indication for a coronectomy did not differ significantly between molars in classes I and II (*p* = 0.892) or between those in classes I and III (*p* = 0.045). The post-hoc analyses of impaction depth showed that the indication for coronectomy was more frequent among third molars in position B (67.4%) compared with molars in position A (54.0%; *p* < 0.001; Table 8). The implication is that for mandibular third molars in position B, the surgeon tended to opt for a coronectomy, and for position A, surgical removal was the choice. The frequency of indication for coronectomy did not differ significantly between molars in positions B and C (*p* = 0.670) or those in positions A and C (*p* = 0.255).

**Table 7:** Post hoc analysis of space between the second molar and the ramus

	Class I and Class II		Class II and Class III		Class I and Class III	
Surgeon’s choice						
Surgical removal	14 (40.0)	291 (41.1)	291 (41.1)	15 (21.4)	14 (40.0)	15 (21.4)
Coronectomy	21 (60.0)	417 (58.9)	417 (58.9)	55 (78.6)	21 (60.0)	55 (78.6)
Total	35 (100)	708 (100)	708 (100)	70 (100)	35 (100)	70 (100)
Chi-square (df)	0.019 (1)		10.469 (1)		4.026 (1)	
*P* value*	0.892		0.001		0.045	

^*^Statistically significant difference at *p* < 0.0167 (Bonferroni correction).

**Table 8:** Post hoc analysis of impaction depth

	Position A and Position B		Position B and Position C		Position A and Position C	
Surgeon’s assessment						
Surgical removal	188 (46.0)	120 (32.6)	120 (32.6)	13 (36.1)	188 (46.0)	13 (36.1)
Coronectomy	221 (54.0)	248 (67.4)	248 (67.4)	23 (63.9)	221 (54.0)	23 (63.9)
Total	409 (100)	368 (100)	368 (100)	36 (100)	368 (100)	36 (100)
Chi-square (df)	14.444 (1)		0.182 (1)		1.297 (1)	
*P* value*	0.0001		0.670		0.255	

^*^Statistically significant difference at *p* < 0.0167 (Bonferroni correction).

## Discussion

In this study, the association between a surgeon’s choice of mandibular third molar coronectomy and the Pell and Gregory or Winter classification system was assessed. Panoramic radiographs were examined from patients whose preoperative radiographs revealed a close relationship between the mandibular third molar and the IAN. Use of the Pell and Gregory classification system was significantly related to the surgeon’s decision to perform a coronectomy (*p* = 0.002). Class III (78.6%) and position B (67.4%) were the most common classifications in cases of coronectomy. The Winter classification system was not associated with the surgeon’s choice of procedure (*p* = 0.425). Following the initial analyses, a post hoc test was performed to probe the origin of statistically significant associations between Pell and Gregory classifications and surgeon assessment. This analysis identified two pairs of factors that were statistically significant: classes II (58.9%) and III (78.6%) and positions A (54.0%) and B (67.4%; both *p* < 0.0167). Other classification pairs were not statistically significant after Bonferroni correction, indicating that the initial significant association was not solely attributable to class III and position B impactions.

To the best of our knowledge, no studies have investigated choice of surgical procedure in association with the Pell and Gregory or Winter classification as evaluated on a preoperative panoramic radiograph. Most previous work using these classification systems in relation to a coronectomy has focused on root migration of mandibular third molars following a coronectomy. A few studies have used these classification systems to assess the diagnostic value of panoramic radiographs in predicting a true relationship between the mandibular third molar and the IAN [16, 17, 19, 20]. In those investigations, the impaction pattern was compared with a gold standard, either a CT or cone-beam CT scan. Other studies have compared the impaction pattern with a more clinical outcome, such as IAN damage or IAN exposure [7, 11, 15, 21], although the surgical procedure was limited only to surgical removal of the mandibular third molar. In the present study, the impaction pattern was compared with the surgeon’s choice of procedure. To limit the possibility of IAN injury, after reviewing CT scans, the surgeon could decide on a coronectomy rather than surgical removal. Although the surgeon’s ultimate assessment was based on CT scan data, the current findings cannot be directly compared with those of earlier studies. Given the absence of directly comparable studies, however, a comparison is given below to contextualize the present findings.

Monaco et al. assessed an association between the Pell and Gregory classification and the topographic relationship between the third molar and the IAN [16, 20], evaluating the accuracy of the radiographic markers by comparing the panoramic radiograph with a CT scan [16]. They found a significant association of the space between the second molar and the ramus (class I/II/III), as seen on the panoramic radiograph, and a true relationship between the third molar and the mandibular canal, as determined by the CT scan (*p* = 0.03). In comparison, in the present study using panoramic radiographs, impaction patterns were compared by the surgeon’s choice of procedure, ultimately based on CT scan data. In cases of a true relationship, class III was most common, a consistency among the findings of these studies. Monaco et al. [16] also noted that as the amount of coverage by the ramus increased, the frequency of a true relationship increased. The present study found a significant association between the surgeon’s decision and the space between the second molar and the ramus (*p* = 0.005). Nunes et al. [20], in contrast, found no significant association of the space between the second molar and the ramus and intimate contact with the mandibular canal (*p* = 0.57). In that study, panoramic radiographs were compared with cone-beam CT scans to evaluate predictive panoramic signs.

Nakagawa et al. [19] and Nunes et al. [20] found no significant association between impaction depth (position A/B/C) and intimate contact with the mandibular canal. However, Monaco et al. [16] reported an association between impaction depth and a true relationship with the IAN (*p* = 0.02), which is again consistent with the current study. The similarities between the study groups in Monaco et al. [16] and the current work could explain the consistency, and both investigations employed radiographic signs as selection criteria, whereas Nunes et al. [20] did not. Worth noting is that these previous studies had sample sizes ranging from 73 to 148 molars [16, 19, 20], whereas the present study used a total of 813 mandibular third molars. As such, any hypothetical inaccuracy in classifying third molars would have affected study outcomes more with a lower sample size.

In the present study, a post hoc analysis revealed that class III (*p* = 0.001) and position B (*p* < 0.001) were primarily responsible for the overall association between the surgeon’s choice and the two categories of the Pell and Gregory classification. Some studies have used bone coverage to define “impaction depth” instead of using the space between the second molar and the ramus according to the Pell and Gregory classification [7, 15, 21]. The term “impaction depth” thus is defined differently in these studies than in the current work, having been used to classify the amount of bone coverage (erupted, partly erupted, unerupted). The results of these studies have suggested that the amount of bone covering the molar correlates with postoperative sensory changes.

In comparison to the other classes and positions within the Pell and Gregory classification, class III and position C can be considered as reflecting higher amounts of bone coverage [16, 19, 20]. According to Carmichael and McGowan [21] and Kipp et al. [15], neurosensory changes are seen most often when the third molar is entirely covered with bone. Valmaseda et al. [7] found that intraosseous impaction was linked to a higher risk of IAN injury, although not significantly so (OR = 2.82, *p* = 0.088). These results suggest that increased bone coverage could lead to more coronectomy indications, although such an association did not emerge in the present study (Table 3, 4, 5).

Use of the Winter classification of a mandibular third molar has led to conflicting results regarding associations with a true relationship with the IAN. Carmichael and McGown [21], Kipp et al. [15], and Nunes et al. have reported such an association. Nakagawa et al. [19] and Valmaseda et al. [7], as well as the current study (*p* = 0.425), however, found none. The observed frequencies for the different inclinations in these studies also are inconsistent, but a bias towards a certain distribution could not be identified. The inclination of an impacted mandibular third molar may have no influence on the surgeon’s decision to perform a coronectomy, as was observed in the present study (Table 6).

This study had some limitations. Only one reviewer evaluated the preoperative panoramic radiographs, although consistency was determined through an analysis using kappa statistics to reflect intra-rater reliability. The kappa values ranged from good to very good, indicating a consistent measurement at the two time points. The validity of this single reviewer, i.e., inter-rater reliability, could not be evaluated in the absence of other reviewers, leaving the possibility that the reviewer was consistently wrong, without a way to verify the accuracy.

For study inclusion, involved molars had to be in a close relationship with the IAN, as indicated when at least one of five radiographic signs associated with increased risk of IAN injury were visible on the preoperative panoramic images. Darkening and narrowing of the root, interruption of the white line, and narrowing and diversion of the canal were all regarded as signs of study eligibility [10, 11, 18]. Most studies used for comparison in the current work focused on a sample of patients who required removal of impacted mandibular third molars, without considering preoperative radiographic signs [7, 15, 20, 21]. No selection criteria related to risk of IAN injury were used in these studies. Not using radiographic signs for selection criteria can affect the distribution of the sample, yielding relatively fewer high-risk patients, but in the present study, only high-risk patients were eligible. This difference may have influenced outcomes in earlier studies, making comparison with the current study more difficult.

A question arises regarding the clinical utility of knowing the extent to which the Pell and Gregory or Winter classification can affect a surgeon’s decision to perform a mandibular coronectomy. Relying only on impaction pattern and foregoing additional 3D imaging would not be advised [22]. However, limiting additional imaging would result in lower radiation exposure and decrease financial costs compared with panoramic imaging [23]. A poor diagnosis, on the other hand, could result in serious complications, such as postoperative neurosensory impairments [8]. Knowing the association of mandibular third molar impaction patterns and the risk for IAN injury could help a clinician determine whether to use additional 3D imaging, supporting decisions about surgical techniques to limit the risk of IAN injury.

In conclusion, this study showed a relationship between the Pell and Gregory classification and a surgeon’s decision to perform a mandibular third molar coronectomy. Coronectomy was preferred over surgical removal more frequently for molars in class III and position B. The Winter classification of mandibular third molars was not associated with the surgeon's choice. Future studies should assess the accuracy of the Pell and Gregory classification in predicting a true relationship with the mandibular canal.

## Data Availability

No datasets were generated or analysed during the current study.
